# Tumor-Targeted Cell-Penetrating Peptides Reveal That Monomethyl Auristatin E Temporally Modulates the Tumor Immune Microenvironment

**DOI:** 10.3390/molecules29235618

**Published:** 2024-11-27

**Authors:** Mahsa Mortaja, Marcus M. Cheng, Alina Ali, Jacqueline Lesperance, Dina V. Hingorani, Mike M. Allevato, Kanika Dhawan, Maria F. Camargo, Rana R. McKay, Stephen R. Adams, J. Silvio Gutkind, Sunil J. Advani

**Affiliations:** 1 Department of Radiation Medicine and Applied Sciences, University of California San Diego, La Jolla, CA 92093, USA; mmortaja@health.ucsd.edu (M.M.); marcus.coujy@gmail.com (M.M.C.); alali@ucsd.edu (A.A.); jlesperance@health.ucsd.edu (J.L.); dhingoraniucsd@gmail.com (D.V.H.); kdhawan@health.ucsd.edu (K.D.); mafe98@gmail.com (M.F.C.); 2 Department of Pharmacology, University of California San Diego, La Jolla, CA 92093, USA; mmallevato@gmail.com (M.M.A.); sadams@health.ucsd.edu (S.R.A.); sgutkind@health.ucsd.edu (J.S.G.); 3 Department of Medicine, University of California San Diego, La Jolla, CA 92093, USA; rmckay@health.ucsd.edu; 4 Moores Cancer Center, UC San Diego, La Jolla, CA 92037, USA

**Keywords:** peptide–drug conjugates, cell-penetrating peptides, anti-tubulins, matrix metalloproteinases

## Abstract

Chemotherapies remain standard therapy for cancers but have limited efficacy and cause significant side effects, highlighting the need for targeted approaches. In the progression of cancer, tumors increase matrix metalloproteinase (MMP) activity. Leveraging and therapeutically redirecting tumor MMPs through activatable cell-penetrating peptide (ACPP) technology offers new approaches for tumor-selective drug delivery and for studying how drug payloads engage the tumor immune microenvironment. ACPPs are biosensing peptides consisting of a drug-conjugated polycationic cell-penetrating peptide masked by an autoinhibitory polyanionic peptide through an interlinking peptide linker. Since tumors overexpress MMPs, ACPP tumor-targeting is achieved using an MMP cleavable linker. Monomethyl auristatin E (MMAE) is a potent anti-tubulin and common drug payload in antibody drug conjugates; however there are limited pre-clinical studies on how this clinically effective drug modulates the interplay of cancer cells and the immune system. Here, we report the versatility of ACPP conjugates in syngeneic murine cancer models and interrogate how MMAE temporally alters the tumor immune microenvironment. We show that cRGD-ACPP-MMAE preferentially delivered MMAE to tumors in murine models. Targeted cRGD-ACPP-MMAE demonstrated anti-tumor kill activity that activated the innate and adaptive arms of the immune system. Understanding how targeted MMAE engages tumors can optimize MMAE tumor kill activity and inform rational combinations with other cancer therapeutics.

## 1. Introduction

Metastasis remains the predominant cause of cancer mortality and is associated with treatment morbidities from non-targeted chemotherapies that diminish a patient’s quality of life. A molecular understanding of biologic pathways that drive tumor progression offers opportunities to develop targeted systemic therapies that selectively kill cancer cells while simultaneously avoiding damage to normal tissues [1]. One strategy for realizing precision cancer therapy involves splitting tumor targeting and cancer killing into two distinct molecular tasks by utilizing carrier vehicles to deliver drug payloads specifically to tumors. Delivery vehicles carry their drug cargoes in a “pro-drug” state that circulates inertly in the vasculature. They preferentially accumulate within tumors, where the bound drug payloads are decoupled from the delivery vehicle. Selective tumor drug delivery can be accomplished by either passive or active targeting mechanisms [2]. Passive targeted carriers lack molecularly defined beacons and instead rely on intrinsic tumor tissue differences. One example of this involves taking advantage of the leaky tumor vasculature as a consequence of dysregulated angiogenesis, which results in the enhanced permeability and retention (EPR) effect utilized by nanoparticles and liposomes [3]. Alternatively, active targeting involves the engagement of the delivery vehicle with defined molecular targets overexpressed in tumors compared to normal tissues. A clinical example of this approach is antibody drug conjugates (ADCs), wherein a tumor-targeting antibody is covalently linked to a drug [4,5]. While ADCs are generating excitement with efficacy in cancer patients, they are limited to subsets of cancers that have the requisite overexpressed receptors. Moreover, ADC efficacy is limited by the resistance and heterogeneity of target receptor expression within a tumor [6].

In the progression of cancers, a matrix metalloproteinase (MMP) remodeling of the extracellular tumor environment is essential for invasion and metastases [7]. The fundamental role that MMPs play in tumor spread has made them a focus for developing new cancer therapies. While inhibitors to MMPs have been developed, pharmacologically blocking MMPs has not proven effective in patients [8,9]. Alternative to enzymatic inhibition, tumor MMP activity can be therapeutically co-opted for targeting drug delivery [10]. An example of this strategy involves cloaking cell-penetrating peptide–drug conjugates with an MMP-sensitive scaffold to create activatable cell-penetrating peptides (ACPPs) [11,12]. ACPPs are bio-sensing peptides that leverage MMP activity for targeted drug delivery. Intact, ACPPs hold the conjugated drug payload in an inaccessible pro-drug state [13]. Engagement with tumors results in extracellular MMP-directed ACPP cleavage and spatially localized deposition of the cell-penetrating peptide–drug conjugate. For translation relevance, drug attachment to ACPPs can utilize identical drug-linker chemistry developed for ADCs [14]. One of the pioneering ADCs was brentuximab–vedotin (Adcetris) [15]. It was constructed by coupling a CD30-targeting antibody (brentuximab) to a monomethyl auristatin E (MMAE) drug payload through the maleimidocaproyl–valine-citrulline–p-aminobenzyloxycarbonyl (MC-VC-PABC) linker [16]. MMAE is an anti-tubulin causing mitotic arrest and cell death in the single nanomolar range [17,18]. While a highly potent cytotoxin, toxicity to normal tissues as a free drug necessitates that MMAE be targeted to tumors for it to have clinical utility [19]. Targeting MMAE to tumors as an alternative to ADC receptor-based therapy may broaden its therapeutic impact.

Within the last decade, paradigm-shifting immune oncology approaches have revolutionized cancer care in patients through immune checkpoint inhibitors [20,21]. However, similar to the clinical experience with ADCs, tumor control from cancer immunotherapies has been limited to subsets of cancer patients. Understanding how the cytotoxic drug payloads of ADC influence the tumor immune microenvironment in pre-clinical cancer models can inform rationale combinations of ADCs with cancer immunotherapies that potentiate anti-tumor immune responses. Unfortunately, there is a paucity of pre-clinical data on ADCs’ modulation of the tumor immune microenvironment [14,22,23]. This is primarily due to species specificity of clinically approved ADCs for human isoforms of receptors that cannot be used in syngeneic murine cancer models. To overcome these hurdles and interrogate how ADC drug payloads influence the tumor–immunity interplay, alternative drug delivery platforms can provide a solution for testing the drug payload components of ADCs in syngeneic murine cancer models.

Here, we report a precision delivery approach for MMAE using tumor-targeted cell-penetrating peptides by conjugating MMAE to ACPPs using identical linker chemistry found in clinically approved ADCs. An advantage of ACPPs is that they can be interchangeably tested in immune-deficient and immune-competent murine cancer models, as opposed to ADCs, which are overwhelmingly restricted to human xenograft models. Spatial in vivo biodistribution studies of ACPPs showed that they are capable of targeting both human xenograft and mouse syngeneic tumors. Therapeutically, we demonstrated that systemically delivered ACPP-MMAE conjugate produced tumor growth delay in syngeneic animal models. Finally, interrogating the temporal modulation of the tumor immune microenvironment revealed that tumor-targeted MMAE engaged both the innate and adaptive arms of the immune system. Taken together, we believe ACPP–drug conjugates can foster the clinical translation of targeted anti-cancer drugs as pre-clinical tool molecules for evaluating ADC drug payloads in immune-competent murine models and for continued clinical development as receptor-independent actively targeted therapeutic delivery vehicles.

## 2. Results

### 2.1. Tumor Matrix Metalloproteinases-Directed Drug Delivery

**Figure 1:**
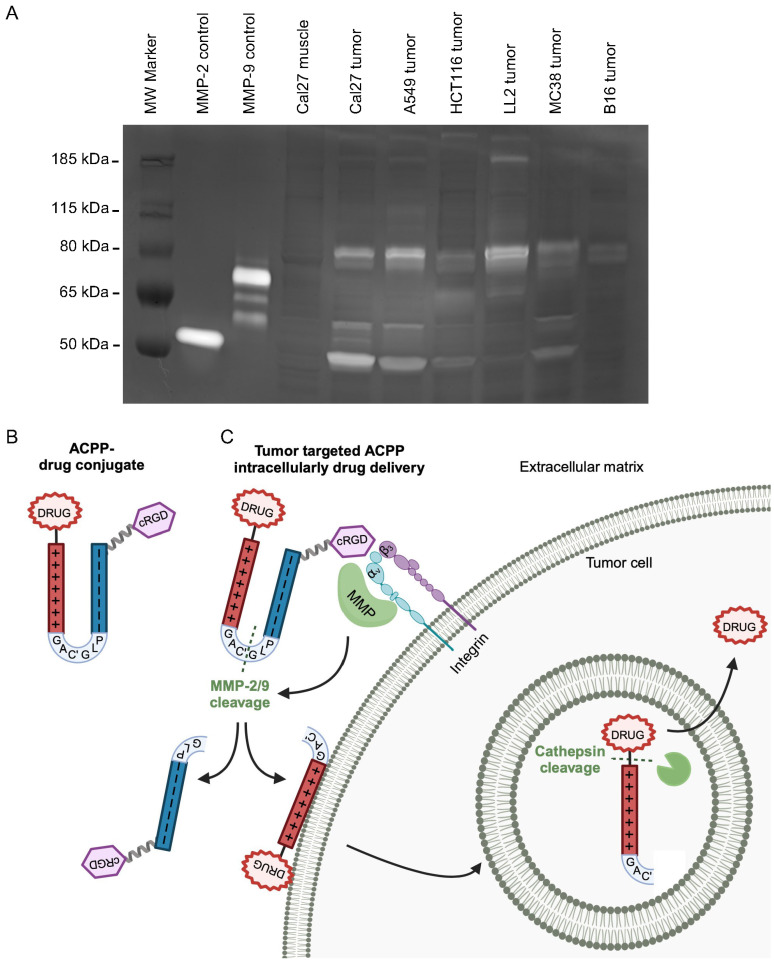
Tissue-targeting cell-penetrating peptides using matrix metalloproteinases. (**A**) Gelatin zymography of human xenograft and syngeneic murine tumors with adjacent normal murine muscle tissue. Molecular weight marker (MW) with control MMP-2 and MMP-9 standard activities in far-left lanes. (**B**) Structural representation of cRGD-ACPP–drug conjugate. Polycationic cell-penetrating peptide (+) and autoinhibitory polyanionic peptide (−) connected by an MMP-2/9 sensitive peptide linker (PLGC(Me)AG, C(Me) denoted C’). (**C**) Schema for activatable cell-penetrating peptide tumor localization. MMP-2/9 cleavage and cathepsin cleavage site indicated by dashed green lines.

Remodeling the extracellular tumor environment by gelatinase MMPs is fundamental to cancer invasion and metastasis. To determine tumor MMP-2 (gelatinase A) and MMP-9 (gelatinase B) activity compared to normal tissue, human xenograft and murine syngeneic tumors were grown in mice and assayed by gelatin zymography (Figure 1A) [24]. Human tumor xenografts originating from head and neck (CAL27), lung (A549), and colorectal (HCT116) cancer cells all demonstrated MMP-2/9 activity. Tumor gelatinase activity was not restricted to cancers of human origin. MMP-2/9 gelatinase activity was also found in murine syngeneic tumors grown in immune-competent mice established from lung (LL2), colorectal (MC38), and melanoma (B16) murine cancer cell lines. Importantly, there was no MMP-2/9 activity in peritumoral muscle tissue excised adjacent to tumors, indicating that gelatinase activity can be leveraged for precision oncology applications.

Cell-penetrating peptides provide a solution to intracellularly deliver bound therapeutic payloads; however, they inherently lack tissue specificity [25,26]. For tumor-targeted delivery, we employed an MMP-2/9-sensitive scaffold that cloaks polycationic cell-penetrating peptide–drug conjugates in an inaccessible state [18,27]. Such ACPPs are constructed using modular architecture that minimally consists of three domains: a polycationic cell-penetrating peptide (nine repeats of D-arginine, r9), a polyanionic autoinhibitory domain (nine repeats of D-glutamic acid, e9), and an intervening MMP-2/9-sensitive peptide linker (PLGC(Me)AG) (Figure 1B). For therapeutic application, a drug payload is conjugated to the cell-penetrating peptide moiety of ACPP. In addition, a pre-targeting cyclic RGD (cRGD) is attached to the polyanionic portion since it has been reported that the hemopexin domain of MMP-2 interacts with α_v_β_3_ integrins [28,29]. Intact, the ACPP–drug conjugate holds the attached drug in an inactive “pro-drug” state due to the charge neutralization of the polycationic cell-penetrating peptide provided by the linked polyanionic peptide. The charge neutralization of the ACPP prevents any electrostatic adhesion of the cell-penetrating peptide–drug conjugate to cell membranes. To validate this, we used Cy5-labeled r9 polycationic cell-penetrating peptide and cRGD-ACPP probes (Figure S1) [13]. The r9 polycationic peptide readily bound murine cancer cells. Cloaking the r9 cell-penetrating peptide in the ACPP scaffold effectively blocked attachment to the same murine cancer cell lines. Following engagement with the extracellular tumor environment, MMP-2/9 cleavage at the intervening PLGC(Me)AG linker within ACPP “activates” the ACPP by removing the spatial inhibition of the polyanionic moiety (Figure 1C). The released polycationic cell-penetrating peptide–drug conjugate then attaches to cell membranes, followed by intracellular uptake into endolysosomes. Protease activity within lysosomes can cleave the drug payload off the cell-penetrating peptide, resulting in tumor-targeted intracellular drug delivery.

### 2.2. Activatable Cell-Penetrating Peptide–Drug Conjugate

**Figure 2:**
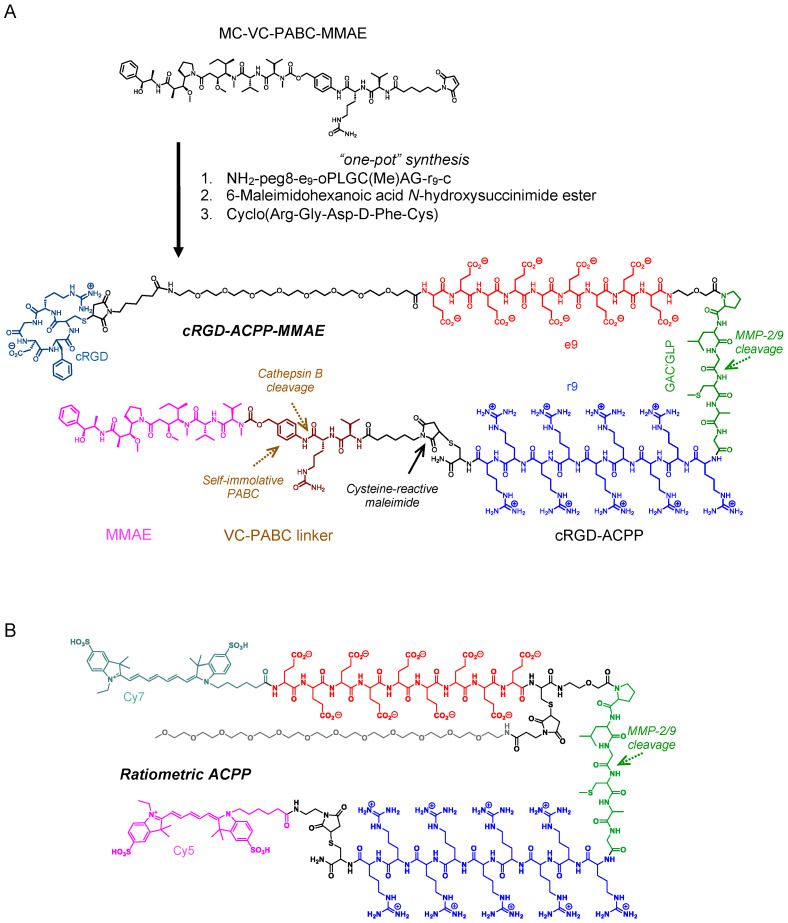
Chemical structures of MMP-sensitive activatable penetrating peptides. (**A**) Synthetic scheme for conjugating monomethyl auristatin E to activatable cell-penetrating peptide. MMAE attached to MC-VC-PABC linker reacted with ACPP and cRGD to yield co-targeted cRGD-ACPP-MMAE. (**B**) Ratiometric ACPP labeled with Cy5 and Cy7 to the polycationic and polyanionic ends, respectively.

For cytotoxic drug payload attachment to ACPP, we have focused on the potent anti-tubulin MMAE given its success as the drug component of clinically effective and approved ADCs. MMAE belongs to a family of auristatins that are synthetic analogues of naturally occurring dolastatin 10 [30]. Dolastatin 10 and derivative auristatins are highly potent drugs that kill cells by blocking tubulin polymerization and resultant G_2_/M cell cycle arrest [19]. To attach MMAE to ACPPs, we utilized the linker chemistry of clinically approved ADCs, i.e., MC-VC-PABC. MMAE coupled to the MC-VC-PABC linker is subsequently attached to the polycationic cell-penetrating peptide through its cysteine-reactive maleimide (Figure 2A). An advantage of the MC-VC-PABC linker for peptide–drug conjugation is that, following internalization into endolysosomes, the intervening valine-citrulline (VC) dipeptide is cleaved by lysosomal cathepsin B [17]. This then results in the self-immolative loss of *p*-iminoquinone methide with CO_2_ and the release of free drug (Figure 1C).

### 2.3. Biodistribution of MMP-Targeted Cell-Penetrating Peptides

**Figure 3:**
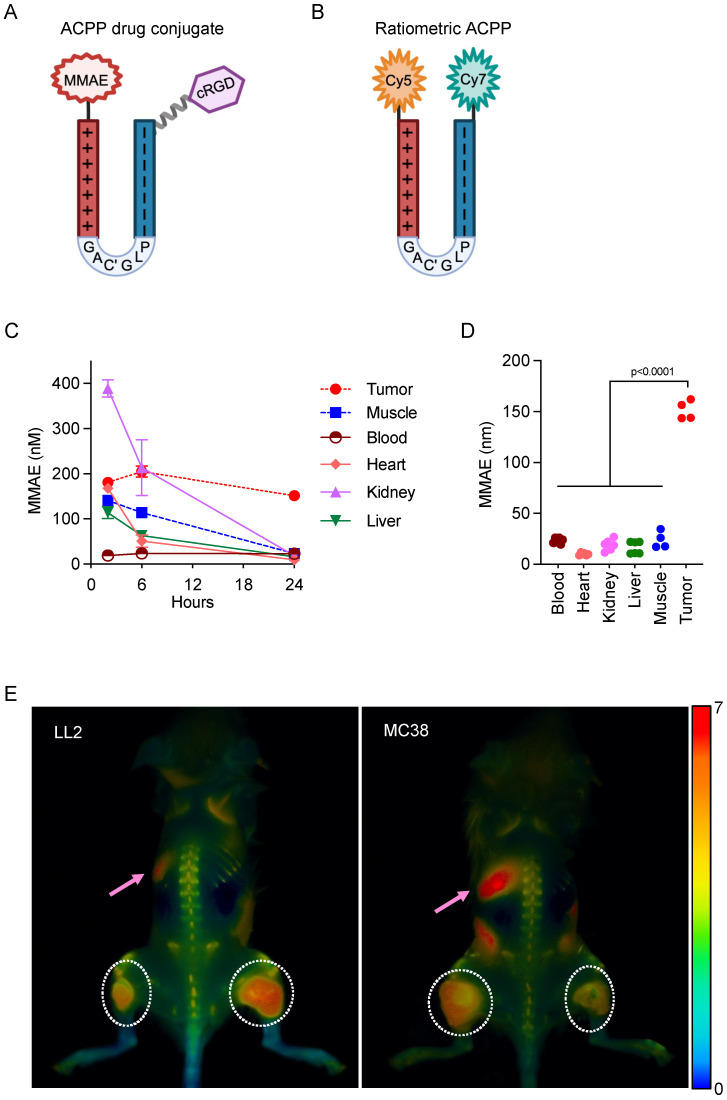
Biodistribution of MMP-guided activable cell-penetrating peptides in vivo. (**A**) Structural representations of activatable cell-penetrating peptide–MMAE conjugate. (**B**) Structural representations of ratiometric activatable cell-penetrating peptide. (**C**) Mice with HCT116 tumor xenografts injected with cRGD-ACPP-MMAE. Tissues harvested at indicated time points and drug concentration determined. Data plotted as mean ± SEM. (**D**) Scatter plot of drug concentration in individual tissues at 24 h post-injection. Statistical significance calculated using one-way ANOVA with Tukey’s multiple comparisons test. (**E**) Mice with syngeneic subcutaneous LL2 or MC38 tumors (white dotted circles indicate bilateral tumor locations) injected IV with ratiometric ACPP. In situ whole-mouse imaging of Cy5 and Cy7 with Cy5:Cy7 emission ratio calculated. Gut auto-fluorescence indicated by pink arrows. Pseudocolor Cy:C7 emission ratio scale bar shown far right.

To determine tissue specificity and the kinetics of ACPP targeting in human and murine tumor model systems, we used complementary approaches, measuring drug concentrations in harvested tissues and non-invasive fluorescence whole-animal imaging. First, the pharmacokinetics of cRGD-ACPP-MMAE drug accumulation were measured in tumor and normal tissues from mice with human HCT116 xenografts. Tumor-bearing mice were intravenously (IV) injected with cRGD-ACPP-MMAE (Figure 3A). Blood, tumor, and normal tissues (heart, kidney, liver, and muscle) were collected 2, 6, and 24 h after IV injection and assayed for MMAE by LC–MS/MS (Figure 3C). All normal tissues showed peak MMAE concentration at the initial time point collected post injection, 2 h. At this time, the kidneys had the highest levels of MMAE, likely due to renal drug excretion. All normal tissues showed a rapid decrease in MMAE concentration after 6 h post-drug delivery. In contrast, tumor MMAE concentration remained relatively constant over 24 h post-injection. To determine the spatial specificity of ACPP drug delivery, normal muscle tissue adjacent to the tumor xenograft was harvested. While peri-tumoral normal muscle tissue showed similar initial MMAE concentrations to more distant normal tissues (i.e., heart and liver) that were comparable to tumors, tumors’ MMAE concentration was higher than the surrounding normal muscle tissue by 6 h. At 24 h post-injection, tumor MMAE was significantly higher than all normal tissues assayed (Figure 3D).

Given the excitement surrounding cancer immunotherapies, pre-clinical testing in syngeneic tumor models with an intact immune system is critical for understanding how therapies modulate tumor immune responses. Since tumors originating from human or mouse cancer cell lines expressed MMP-2 and MMP-9 (Figure 1A), we tested the spatial specificity of ACPP cleavage and localization in syngeneic murine tumors grown in immune-competent mice. A ratiometric ACPP probe was synthesized by attaching a Cy5 far red fluorescent donor to the polycationic cell-penetrating peptide end and a Cy7 near infrared fluorescent acceptor to the polyanionic peptide end (Figure 2B) [31]. While ratiometric ACPP is intact, Cy7 re-emission is favored when excited with Cy5 excitation wavelengths, resulting in a low Cy5:Cy7 emission ratio (Figure 3B). However, when the MMP-2/9-sensitive PLGC(Me)AG linker of ratiometric ACPP is cleaved, Cy5 emission from the labeled polycationic cell-penetrating peptide is no longer quenched, resulting in increased Cy5:Cy7 emission ratio. Immune-competent mice with syngeneic LL2 or MC38 tumors were IV injected with ratiometric ACPP and imaged 90 min later. In both syngeneic murine tumor models, increased Cy5:Cy7 emission ratio spatially localized to the bilateral subcutaneous tumors in the hindlimbs, indicative of the tumor-selective cleavage of ACPP (Figure 3E). Gut autofluorescence is indicated by pink arrows. Taken together, these results highlight the spatial specificity with which cell-penetrating peptides cloaked in an MMP-2/9-sensitive scaffold as an ACPP can target conjugated drug payloads to tumors in commonly used pre-clinical xenograft and syngeneic murine cancer models.

### 2.4. Therapeutic Activity of cRGD-ACPP-MMAE

**Figure 4:**
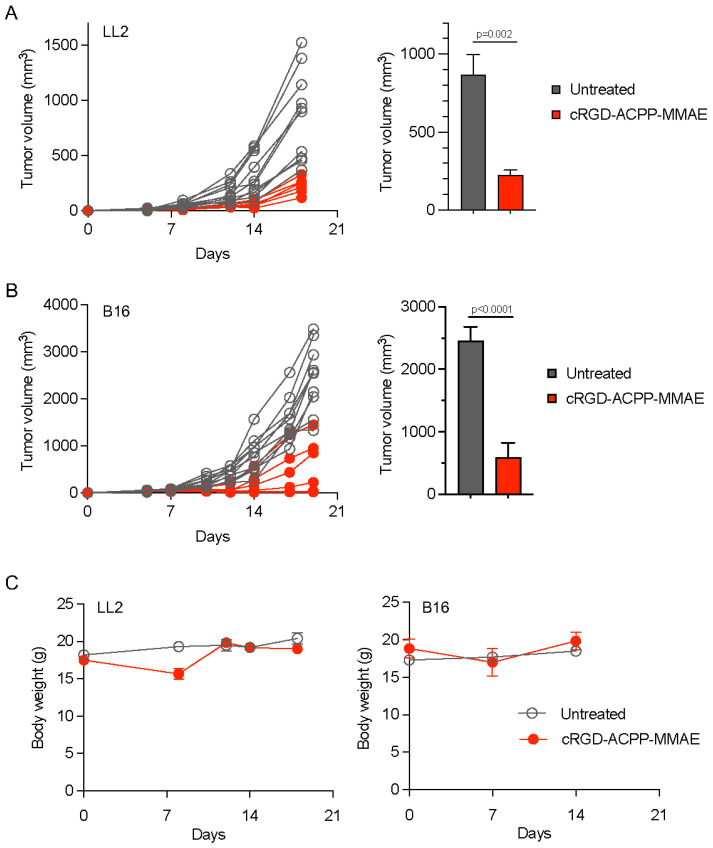
Anti-tumor efficacy of cRGD-ACPP-MMAE. (**A**) Mice bearing LL2 tumors IV injected with vehicle (untreated) or cRGD-ACPP-MMAE (20 nmoles on days 4 and 6). Individual tumor volumes plotted, left panel. Average tumors volumes on day 18 plotted as mean ± SEM, right panel (n = 10 for untreated, n = 6 for cRGD-ACPP-MMAE). Statistical significance calculated using two-tailed *t* testing. (**B**) Mice bearing B16 tumors IV injected with vehicle (untreated) or cRGD-ACPP-MMAE (20 nmoles on days 3 and 5). Individual tumor volumes plotted, left panel. Average tumors volumes on day 19 plotted as mean ± SEM, right panel (n = 10 for untreated, n = 6 for cRGD-ACPP-MMAE). Statistical significance calculated using two-tailed *t* testing. (**C**) Mouse body weights of mice from experiments in (**A**,**B**), plotted as mean fractional body weight ± SEM.

Next, we evaluated the efficacy of cell-penetrating peptide–MMAE conjugates in syngeneic tumor models. Immune-competent C57BL6 mice bearing LL2 tumors were treated with IV cRGD-ACPP-MMAE. Compared to untreated tumors, cRGD-ACPP-MMAE resulted in significant delayed tumor growth (Figure 4A). By day 18, cRGD-ACPP-MMAE-treated mice had an average tumor volume of 227 ± 30 mm^3^, compared to 868.2 ± 127.7 mm^3^ for untreated mice. To corroborate these results, we tested the efficacy of tumor-targeted MMAE in the B16 murine melanoma model. Validating our findings with LL2 tumors, mice treated with IV cRGD-ACPP-MMAE demonstrated tumor growth delay compared to untreated mice (Figure 4B). By day 19, cRGD-ACPP-MMAE-treated mice had an average tumor volume of 588.2 ± 238.2 mm^3^, compared to 2456.7 ± 223.2 mm^3^ for untreated mice. To assess the safety and tolerability of systemically administered ACPP-MMAE conjugate, mice weights were measured weekly in the two therapy studies above. Mouse body weights showed no significant difference between control or cRGD-ACPP-MMAE-treated mice (Figure 4C).

### 2.5. Tumor Immune Microenvironment Modulation by MMAE

**Figure 5:**
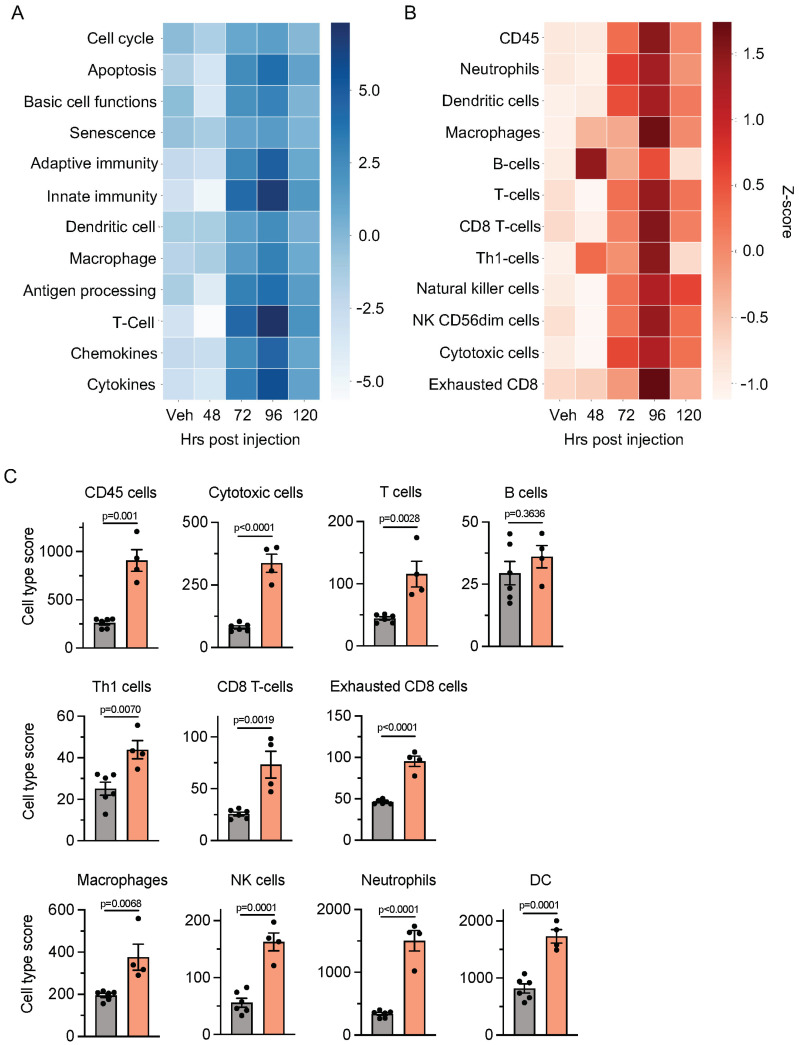
MMAE-induced temporal changes in the tumor immune microenvironment. Mice with B16 tumors treated with IV cRGD-ACPP-MMAE. Tumors harvested at time 48–120 h post-drug injection and analyzed using NanoString nCounter PanCancer Mouse Immune Profiling panel. Heatmap depicts the Z-score of immune signatures for biological pathways (**A**) and individual immune cell types (**B**). (**C**) Individual immune cell type abundance at 96 h after the injection of cRGD-ACPP-MMAE. Data plotted as scatter plot with mean ± SEM. Statistical significance calculated using two-tailed *t* testing.

Finally, we determined if cRGD-ACPP-MMAE-altered tumor immune infiltration occurred. C57BL6 mice bearing B16 tumors were treated with IV cRGD-ACPP-MMAE. Tumors were harvested at 48, 72, 96, and 120 h post-injection. To comprehensively interrogate if targeted MMAE modulated the tumor immune microenvironment, we assayed tumors by the NanoString PanCancer Mouse Immune Profiling gene expression platform [32,33]. We first analyzed temporal changes induced by cRGD-ACPP-MMAE on major cellular and immunologic pathways (Figure 5A). At 48 h, IV injected cRGD-ACCP-MMAE produced minimal changes in tumors compared to vehicle-treated mice. Interestingly, cRGD-ACPP-MMAE altered multiple pathways after 72 h that peaked 96 h post-drug delivery. The largest changes were observed in pathways involving innate immunity and T-cells. This response showed temporal kinetics with the time of drug administration since, by 120 h post-injection, MMAE-driven alterations in the tumor immune microenvironment decreased towards the baseline of untreated tumors. Next, we focused on the temporal changes induced by MMAE on specific immune cell types (Figure 5B). Concordant with our pathway-based analysis, all immune cell types peaked at 96 h post-cRGD-ACPP-MMAE injection compared to untreated tumors. The sole exception was B cells that peaked earlier at 48 h. Finally, we measured changes to individual immune cell types driven by MMAE at peak effect in the tumors, i.e., 96 h post-injection (Figure 5C). By a broad pan immune cell marker (CD45+), MMAE-conjugate-treated mice showed a 3.5-fold increase in tumor-infiltrating immune cells. Given that pathway-based gene clustering demonstrated that the innate and adaptive/T-cell immunity pathways had the largest increases with MMAE-conjugate treatment (Figure 5A), we interrogated individual immune cell types involved in these two arms of the immune system at 96 h post-cRGD-ACPP-MMAE delivery. Innate immunity is mediated in part through macrophages, neutrophils, dendritic cells, and NK cells [34,35]. These immune cell types all showed a significant increase within the tumor immune microenvironment of MMAE-treated mice compared to untreated controls. The adaptive immunity arm includes cytotoxic T-cells, B-cells, and T-helper cells. Interestingly, cytotoxic T-cells and Th1 cells were significantly increased in tumors from MMAE-treated mice. However, B-cells showed no statistically significant difference compared to untreated tumors. Characterizing T-cells more deeply revealed that, while CD8+ T-cells increased following MMAE treatment, there was also a concomitant increase in exhausted CD8+ T-cells. Overall, the largest relative changes in MMAE-driven tumor immune infiltration occurred in neutrophils (innate immunity, 4.5-fold) and cytotoxic T-cells (adaptive immunity, 4.2-fold) relative to untreated tumors.

## 3. Discussion

The targeted delivery of potent cytotoxic chemotherapies continues to pose therapeutic challenges in the era of precision medicine [36]. In these studies, we evaluated ACPP–drug conjugates in preclinical syngeneic cancer models grown in immune-competent mice, which are essential to interrogate how cancer therapies modulate tumor immune responses, which can then inform rational integration with cancer immunotherapies. Our ACPP is designed with an MMP-2/9-sensitive scaffold that results in the selective cleavage and delivery of the cloaked cell-penetrating peptide–drug conjugate moiety [12,18]. MMPs are integral to remodeling the tumors and play a critical role in cancer progression, invasion, and metastasis [7,24]. In contrast to pharmacologic inhibition approaches, ACPPs leverage the elevated MMP enzymatic activity of tumors to spatially localize systemically administered cell-penetrating peptide–drug conjugates to tumors. MMP-2 and MMP-9 are broadly expressed in the tumors not only of different histologies but also from different species. By both indirect non-invasive imaging and direct tissue drug measurement, ACPPs accumulated in tumors. The tumor-targeted delivery of MMAE produced tumor regression in highly aggressive syngeneic tumor models. Interestingly, MMAE temporally modulated tumor immune infiltration, which peaked 4 days after systemic drug delivery and then began to recede.

ADCs have emerged as a powerful way to target cytotoxins to tumors in a receptor-restricted fashion [4,37]. The most common clinical drug payloads of ADCs fall into two classes: (1) anti-tubulins (auristatins, maytansinoids) and (2) topoisomerase I inhibitors (SN-38, exatecans) [38]. These drug payloads must be structurally amenable to peptide linkage and also be highly potent since ADC uptake is limited by cell membrane receptor density. MMAE fits these characteristics and is the most common ADC drug payload [5]. Importantly, MMAE is the drug payload of three unique FDA approved ADCs that include brentuximab–vedotin (Adcetris), enfortumab–vedotin (Padcev), and tisotumab–vedotin (Tivdak), targeting CD30, Nectin-4, and tissue factor receptors, respectively [15,39,40]. The linker chemistry for synthesizing these three ADCs is MC-VC-PABC [16]. ADCs have had limited oncologic applications due to targeted receptors being expressed in only certain subsets of cancer patients [5]. Moreover, tumor heterogeneity can result in varied receptor expression within the same patient and therapy resistance [6]. Cancer cells that have low receptor expression can evade being targeted by ADCs and lead to treatment failure. ACPPs offer a solution to this clinically significant issue by exploiting the intrinsically elevated MMP activity of cancers as a localizing beacon for active targeted drug delivery. For translational relevance, we synthesized ACPP–drug conjugates by coupling the same MC-VC-PABC-MMAE drug-linker moiety found in clinically approved ADCs to ACPPs.

Within the last decade, immunotherapy has emerged as a new pillar of cancer therapy with the success of immune checkpoint inhibitors that inhibit CTLA-4, PD-1, and PD-L1 [20,21]. However, as with ADCs, the benefits of cancer immunotherapies are limited to subsets of patients. There is tremendous interest in improving immune checkpoint inhibitor responses to wider patient populations by testing them in combination with cytotoxic cancer therapies [41,42,43,44]. Excitingly, a recent clinical trial in metastatic bladder cancer showed that the combination of the ADC enfortumab–vedotin with anti-PD-1 immune checkpoint inhibitor pembrolizumab improved patient outcomes [45,46]. Relevant to our current studies, the drug payload of enfortumab–vedotin is MMAE. Unfortunately, the pre-clinical testing of MMAE’s effects on the tumor immune microenvironment or in combination with cancer therapies in syngeneic murine models is limited. This is largely due to the specificity of ADCs for human isoforms of receptors and also the lack of robust receptor-driven syngeneic murine cancer models. Our ACPP-MMAE tool molecule provides a solution to these issues since it can be interchangeably tested in human xenograft tumors grown in immune-deficient mice or murine syngeneic tumors grown in immune-competent mice. We found that tumor-targeted MMAE engaged both the adaptive and innate arms of the immune system. Moreover, the temporal response by individual immune cell types suggests that the optimal sequencing of MMAE with cancer therapies may potentiate anti-tumor immune responses.

In summary, our ACPP scaffold redirects tumorigenic MMP protease activity to achieve enzyme-amplified drug delivery by unmasking the cell-penetrating peptide–drug conjugate. By harnessing tumor extracellular proteases, ACPPs function as biosensing probes that identify and selectively release bound drug within tumors while avoiding normal tissues, thereby widening the therapeutic index of the drug payload. The ACPP platform offers an alternative active targeting delivery vehicle to receptor-directed ADCs. From a drug development and translational perspective, since ADCs and ACPPs can use identical linker-drug chemistry for drug attachment, ACPPs offer a versatile pre-clinical tool to evaluate the anti-tumor efficacy of cytotoxic drugs in ADC development. While we have focused on the anti-cancer cytotoxic drug MMAE, other classes of drug payloads have been conjugated to ACPPs, including immunomodulators and DNA damage repair inhibitors [13,47]. Extending beyond oncologic applications, ACPP’s modular architecture allows for alterations of the intervening peptide linker to sequences cleaved by alternative proteases upregulated in other disease processes that can then be preferentially targeted with relevant disease-modifying drugs attached to the cloaked polycationic cell-penetrating peptide portion of ACPPs.

## 4. Materials and Methods

### 4.1. Cells

Human head and neck CAL27, lung A549, colorectal HCT116, murine lung LL2, and melanoma B16 cancer cell lines were obtained from American Type Culture Collection (Manassas, VA, USA). Murine colorectal MC38 cancer cell line was obtained from Kerafast (Shirley, MA, USA). CAL27, A549, HCT116, LL2, and B16 cells were cultured in DMEM (Gibco, Waltham, MA, USA) supplemented with 10% FBS (Omega Scientific, Tarzana, CA, USA). MC38 cells were cultured in DMEM supplemented with 10% FBS, 1mM sodium pyruvate (Gibco), 1% non-essential amino acids (Gibco), and 10mM HEPES (Gibco). On initial receipt, cell lines were expanded, and low-passage stocks were cryopreserved without further authentication testing. Cells were passaged 2 times per week and used for 4–6 weeks, after which a new stock vial was thawed. Cells were routinely tested for mycoplasma by PCR, including testing prior to cell implantation in mice for tumor experiments.

### 4.2. Synthesis of Activatable Cell-Penetrating Peptides

cRGD-ACPP-MMAE was synthesized as previously described [14]. Base ACPP with H_2_N-peg8-e_9_-oPLGC(Me)AG-r_9_-c-CONH_2_ was made using regular solid phase Fmoc peptide synthesis, where lowercase letters refer to D-amino acids, peg8 refers to H_2_N-PEG8-propionic acid, o-denotes 5-amino-3-oxopentanoyl (a short hydrophilic spacer), C(Me) denotes S-methylcysteine, and the final CONH_2_ indicates C-terminal amide. The peptide was cleaved from the resin by treating it with mixtures containing 92% trifluoroacetic acid (TFA), 2% thioanisole, 2% water, and 4% triisopropylsilane (TIPS) for 4 h under N_2_ atmosphere and filtered. This filtrate was concentrated and then precipitated by the addition of ice-cold 50% hexanes in ethyl acetate mixture. Centrifugation was performed to isolate the precipitate that was dried under vacuum. The peptide was dissolved in DMSO and purified by HPLC (Agilent, Santa Clara, CA, USA) using 5–55% acetonitrile in water and 0.05% TFA over a period of 25 min at 3.5 mL/min flow rate. The purified product was dried using lyophilization. ES–MS found 622.8 (M^+^ + 6H^+^), 746.7 (M^+^ + 5H^+^), 932.0 (M^+^ + 4H^+^), 1242.4 (M^+^ + 3H^+^), deconvolved to 3724.9 (M^+^ + H^+^), calculated for C_147_H_259_N_55_O_54_S_2_, 3723.87. H_2_N-peg8-e_9_-oPLGC(Me)AG-r_9_-c-CONH_2_ (10·TFA salt, 9.90 mg, 2.0 μmol) dissolved in dry DMSO (200 μL) was added to MC-VC-PABC-MMAE (2.70 mg, 2.0 μmol), and N-methyl morpholine (2.2 μL, 20 μmol) was added with mixing. LC–MS indicated complete reaction after 30 min to give a single product; ES–MS found 841.3 (M^+^ + 6H^+^), 1009.1 (M^+^ + 5H^+^), 1261.2 (M^+^ + 4H^+^), 1681.5 (M^+^ + 3H^+^), deconvolved to 5041.0, calculated for C_215_H_364_N_66_O_69_S_2_, 5041.8, which was used without further purification. A solution of 6-maleimidocaproic acid N-hydroxysuccinimide ester (Sigma-Aldrich, St. Louis, MO, USA; 20 μL of 100 mM in dry DMSO, 2.0 μmol) was added to the reaction mixture and kept at room temperature for 4 days until LC–MS showed that reaction was complete. ES–MS found 1049.9 (M^+^ + 5H^+^), 1309.5 (M^+^ + 4H^+^), 1745.0 (M^+^ + 3H^+^), deconvolved to 5233.8, calculated for C_225_H_375_N_67_O_72_S_2_, 5235.0. Cyclo(RGD)fC (Peptides International, Louisville, KY, USA, 1.5 mg, 2.6 μmol) dissolved in dry DMSO (100 μL) was added and mixed. LC–MS indicated complete reaction after 30 min to yield final product, and the reaction was quenched with acetic acid (50 μL), separated by HPLC, and lyophilized to give cyclo(RGD)fc-MC-HN-peg8-e_9_-oPLGC(Me)AG-r_9_-c-(MC-VC-PABC-MMAE)-CONH_2_ as a white powder, yield, 7.67 mg (56%) with purity 99%. ES–MS found 831.5 (M^+^ + 7H^+^), 969.8 (M^+^ + 6H^+^), 1163.6 (M^+^ + 5H^+^), deconvolved to 5812.5, calculated for C_249_H_409_N_75_O_79_S_3_, 5813.6. HPLC and a mass spectrometry analysis of cRGD-ACPP-MMAE are shown in Figure S2. Ratiometric activatable cell-penetrating peptide was synthesized as previously described [13]. Peptides were lyophilized and stored as a powder at −20 °C.

### 4.3. Gelatin Zymography Assays

All animal work was performed in compliance with the University of California San Diego Institutional Animal Care and Use Committee. For human xenograft tumors, 6-week-old female athymic nu/nu mice (University of California San Diego Animal Care Program) were injected subcutaneously into the hindlimb region with human CAL27, A549, or HCT116 cells in 100 µL of a 1:1 Growth Factor Reduced Matrigel (BD) and PBS solution. For murine syngeneic tumors, 6-week-old female C57BL/6 albino mice (Jackson Labs) were injected with B16, MC38, or LL/2 cells subcutaneously in 100 µL of 1:1 Growth Factor Reduced Matrigel (BD) and PBS solution. When tumors were palpable, tumor and peri-tumoral muscle were harvested and frozen in liquid nitrogen. Frozen samples were pulverized with mortar and pestle, lysed with NP-40 lysis buffer (25 mM Tris-HCl, pH 7.5, 100 mM NaCl, 1% NP-40, protease inhibitor (Roche Diagnostics, Mannheim, Germany)), homogenized by passing them through a syringe, centrifuged at 16,000× *g* for 10 min at 4 °C, supernatant collected, and protein concentration measured. Then, 2× Tris Glycine SDS Sample Buffer was added to samples and equivalent amounts of total protein were loaded onto 10% zymogram gelatin gels (Novex, Waltham, MA, USA). Gels were developed using Novex zymogram renaturing and developing buffers (10×) and stained with SimplyBlue Safestain (Invitrogen, Waltham, MA, USA). Purified MMP-2 and MMP-9 were loaded to identify gelatinase activities.

### 4.4. Tissue Drug Measurement

Mice with established subcutaneous HCT116 tumors were intravenously injected through the tail vein with 10 nmoles of cRGD-ACPP-MMAE. Tumors, blood, and normal tissues were harvested at indicated time points and homogenized in 10 volumes of PBS with a point sonicator (amplitude range 5–15%). Homogenates were centrifuged (14 g, 10 min) with supernatants collected and diluted 2-fold by the addition of 2% acetic acid in acetonitrile, followed by centrifugation (14× *g*, 10 min). MMAE concentration was measured by using LC–MS/MS with a Luna-2 C18 column (Phenomenex, Torrance, CA, USA) and Agilent Trap XCT mass spectrophotometer (Agilent), with extracted ion currents at 686.4 and 506.4 integrated and combined to improve sensitivity. Ion currents were fitted to a standard curve to determine tissue drug concentrations.

### 4.5. In Vivo Optical Imaging of Fluorescently Labeled Ratiometric ACPP

LL2 or MC38 tumor cells were subcutaneously implanted into the bilateral hindlimbs of 6-week-old C57BL/6 albino mice. When tumors were palpable, mice were anesthetized (1:1 mixture of 100 mg/mL of ketamine and 5 mg/mL of midazolam) and IV injected through the tail vein with 10 nmoles of Cy5- and Cy7-labeled ratiometric ACPP. At 90 min after injection, mice were sacrificed and whole mouse was imaged using the Maestro small-animal imager (CRI, Caliper Life Sciences, Hopkinton, MA, USA). Imaging was performed after skin removal to decrease autofluorescence and scattering. The acquisition parameters were excitation filter 607/36 nm, scanned across a wavelength of 640 nm to 840 nm, emission filter 633 LP. The in vivo images were analyzed with custom software for generating Cy5/Cy7 ratiometric pseudocolor images. Exposure time for fluorescence capture for tumor models was as follows: LL2 for 800 ms, MC38 for 300 ms.

### 4.6. In Vivo Tumor Therapy Studies

LL2 and B16 tumor cells were injected subcutaneously into the hindlimbs of 6-week-old female C57BL/6 albino mice in 100 µL of 1:1 Growth Factor Reduced Matrigel (BD) and PBS solution. Tumors were injected with vehicle or cRGD-ACPP-MMAE, 20 nmoles given on every other day, for an identical total dose of 40 nmoles peptide–drug conjugate per mouse. Tumor volumes were measured and calculated using the formula as ½ × Length × Width^2^. Body weights were measured weekly to assess general animal health.

### 4.7. NanoString Analysis

RNA was isolated from tumors and comprehensive immune profiling was performed using the NanoString (Seattle, WA, USA) nCounter PanCancer Mouse Immune Profiling gene expression platform. The Advanced Analysis module of the nSolver software (NanoString, version 4.0) was used to analyze genes associated with listed biological processes or immune cells in tumors and given a Z-score. Gene set analysis was conducted with calculated global significance scores and directed significance scores for treated cells. The analysis of immune cell-associated genes in tumors was performed using the Advanced Analysis module of the nSolver 4.0 software. Cell type scores were calculated using the Cell Type Profiling module, which quantifies immune cell populations based on marker gene expression data. Pathway scores were computed via the Pathway Scoring module, summarizing gene activity within specific pathways. Both cell type and pathway scores were presented as raw scores and Z-scores, reflecting variations from the mean across samples.

### 4.8. Statistical Analysis

Ordinary one-way ANOVA with Tukey’s multiple comparisons testing was performed for biodistribution experiments (Figure 3D. Unpaired 2-sided *t* tests were performed for murine tumor regression studies and NanoString gene expression analysis (Figure 4A,B and Figure 5C). All statistical analyses were performed using Prism (GraphPad Software, Boston, MA, USA, version 10.0.3).

## Data Availability

All data reported in this work are available upon reasonable request to the corresponding author.

## Supplementary Materials

The following supporting information can be downloaded at: https://www.mdpi.com/article/10.3390/molecules29235618/s1, Figure S1. Cell binding of naked and ACPP cloaked polycationic cell penetrating peptides. Figure S2. HPLC and mass spectrograms of peptides.
