# Supplementary material for: Tumor-Targeted Cell-Penetrating Peptides Reveal That Monomethyl Auristatin E Temporally Modulates the Tumor Immune Microenvironment
Source: Molecules. 2024 Nov 27;29(23):5618. doi: 10.3390/molecules29235618 (PMC11643828; doi:10.3390/molecules29235618)

Supplementary Information:

Tumor targeted cell penetrating peptides reveal monomethyl auristatin E temporally modulates the tumor immune microenvironment

Mortaja M, et al

Figure S1. Cell binding of naked and ACPP cloaked polycationic cell penetrating peptides. (**A**) Chemical structure of Cy5 labeled r9 cell penetrating peptide. (**B**) Chemical structure of Cy5 labeled cRGD-ACPP. (**C**) Structural representations of Cy5 labeled r9 cell penetrating peptide and cRGD-ACPP. (**D**) Murine cancer cells were exposed to 1 μM Cy5 labeled r9 cell penetrating peptide or cRGD-ACPP probe for 2 hours, washed, fixed and DAPI stained. Cells imaged for Cy5 fluorescence of bound cell penetrating peptide probes (Magenta). Nuclei imaged by DAPI (Blue).

Figure S2. HPLC and mass spectrograms of peptides. (**A**) Starting peg8-ACPP peptide. (**B**) Reaction mixture of peg8-ACPP with MC-VC-PABC-MMAE. (**C**) Reaction mixture of peg8-ACPP-MC-VC-PABC-MMAE with MC-Osu. (**D**) Purified cRGD-ACPP-MMAE. Peaks at 3-10 minutes at 215nm are from DMSO solvent.

Figure S1

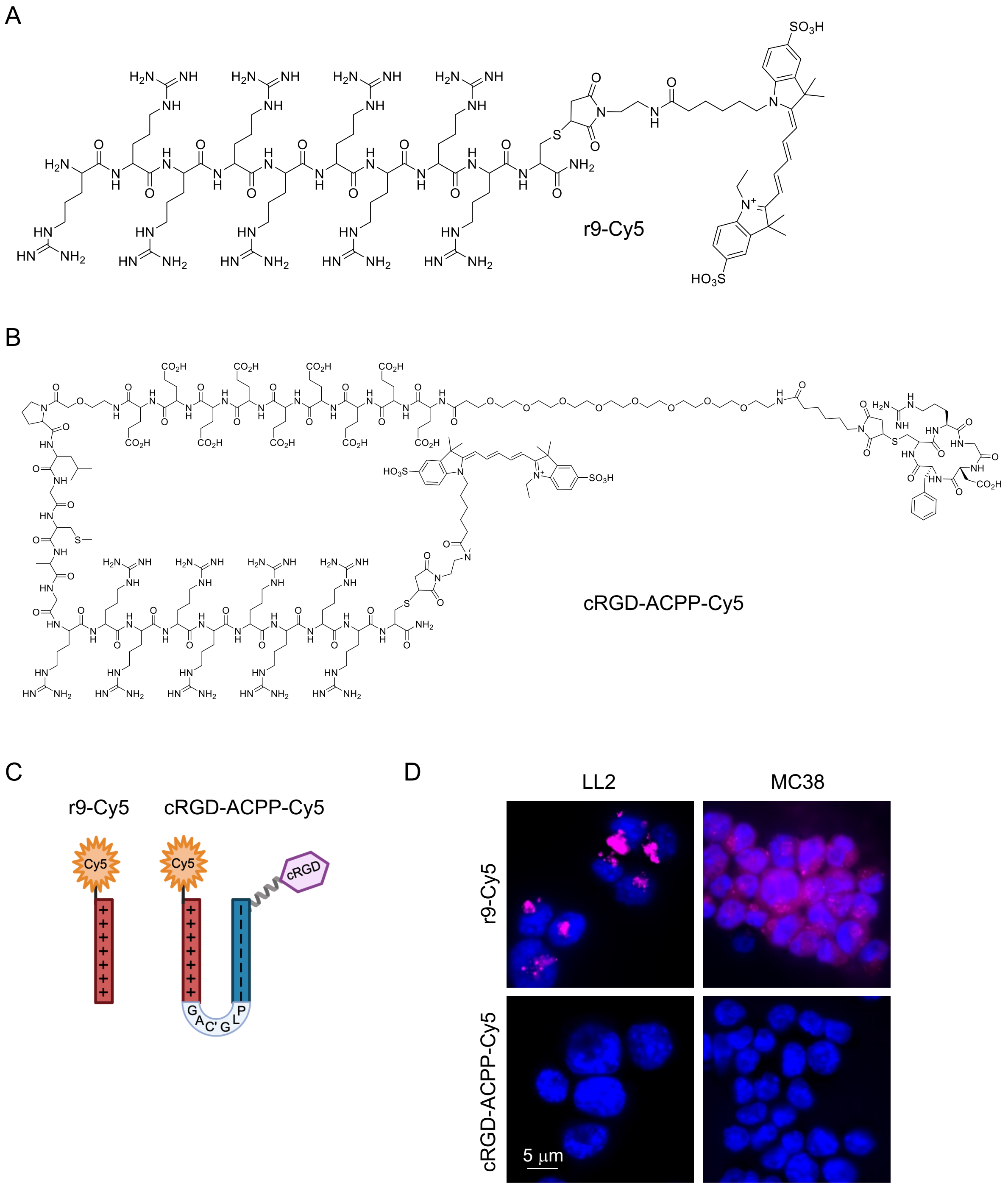
A
r9-Cy5
B
cRGD-ACPP-Cy5
C
r9-Cy5
cRGD-ACPP-Cy5
Cy5
cRGD
D
LL2
MC38
r9-Cy5
cRGD-ACPP-Cy5
5 µm

Figure S2

A

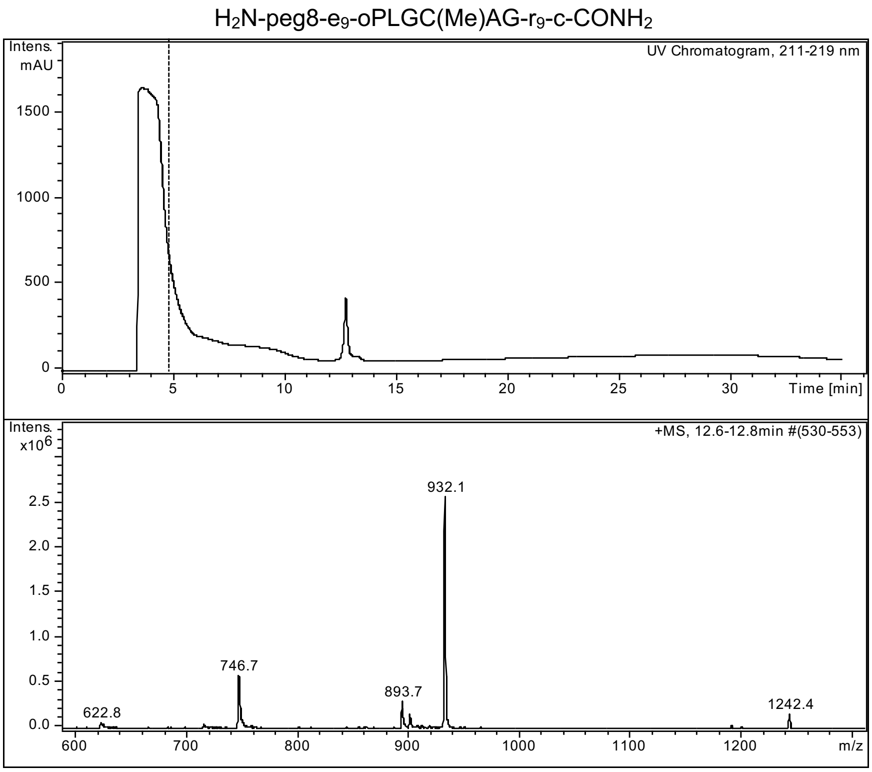

H2N-peg8-e9-oPLGC(Me)AG-r9-c-CONH2
Intens. mAU
UV Chromatogram, 211-219 nm
Time [min]
Intens. x10^6
+MS, 12.6-12.8min #(530-553)
932.1
746.7
893.7
622.8
1242.4
m/z

B

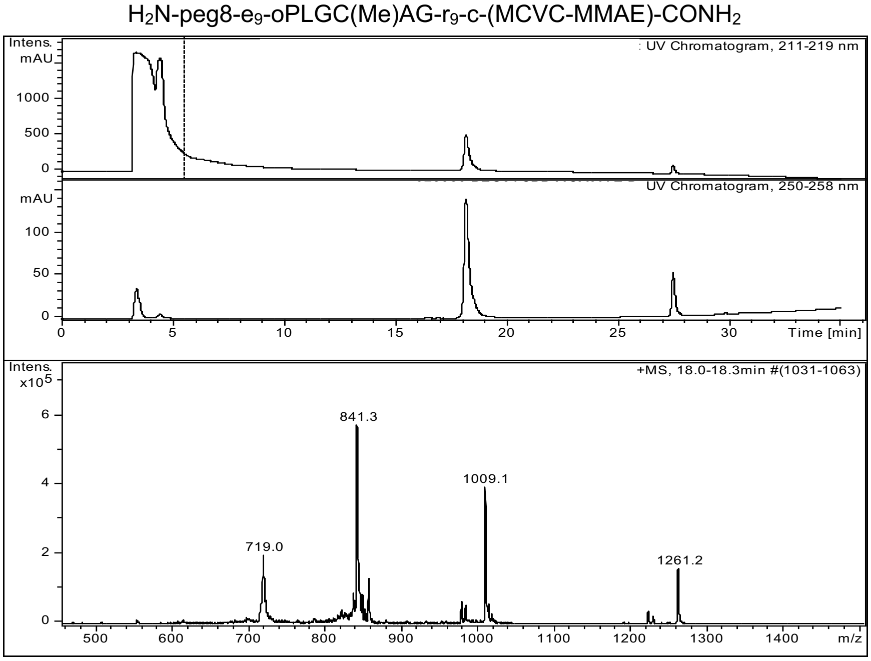

H2N-peg8-e9-oPLGC(Me)AG-r9-c-(MCVC-MMAE)-CONH2
Intens. mAU
UV Chromatogram, 211-219 nm
mAU
UV Chromatogram, 250-258 nm
Time [min]
Intens. x10^5
+MS, 18.0-18.3min #(1031-1063)
841.3
1009.1
719.0
1261.2
m/z

Figure S2

C

MC-CONH-peg8-e9-oPLGC(Me)AG-r9-c-(MCVC-MMAE)-CONH2

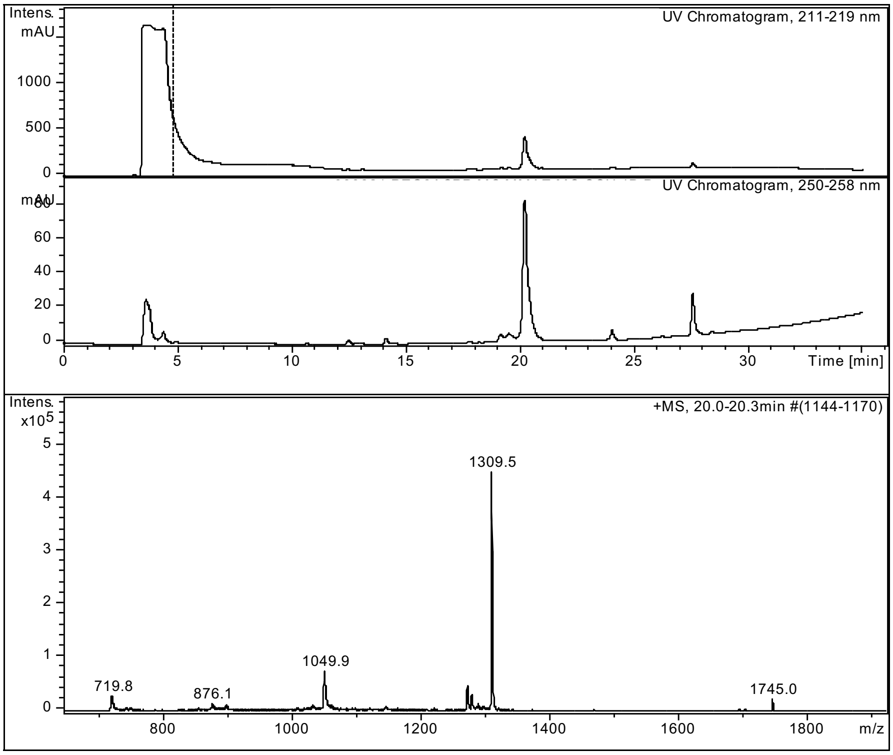

D

Cyclo(RGD)-fC-MC-CONH-peg8-e9-oPLGC(Me)AG-r9-c-(MCVC-MMAE)-CONH2

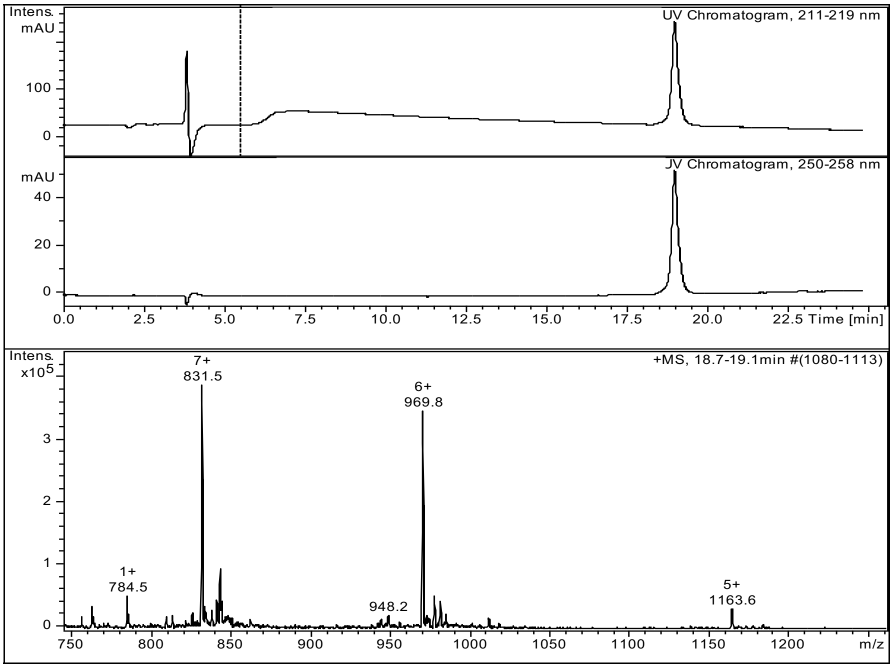

Supplement: Supplementary file 1 [file molecules-29-05618-s001.zip › molecules-3319189-supplementary.pdf]
